# Headache Management in Military Primary Care: Findings from a Nationwide Cross-Sectional Study

**DOI:** 10.3390/jcm14134497

**Published:** 2025-06-25

**Authors:** Carl H. Göbel, Ursula Müller, Hanno Witte, Katja Heinze-Kuhn, Axel Heinze, Anna Cirkel, Hartmut Göbel

**Affiliations:** 1 Kiel Migraine and Headache Center, 24149 Kiel, Germany; ursula-mueller@email.de (U.M.); khk@schmerzklinik.de (K.H.-K.); heinze@schmerzklinik.de (A.H.); anna.cirkel@uni-luebeck.de (A.C.); hg@schmerzklinik.de (H.G.); 2 Department of Neurology, University Hospital Schleswig-Holstein, Campus Kiel, 24149 Kiel, Germany; 3 Department of Neurology, Bundeswehr Hospital Ulm, 89081 Ulm, Germany; 4 Department of Internal Medicine, Bundeswehr Hospital Ulm, 89081 Ulm, Germany; hanno.witte@uni-ulm.de; 5 Department of Neurology, University Hospital Schleswig-Holstein, Campus Lübeck, 23562 Lübeck, Germany

**Keywords:** migraine, headache disorders, military medicine, primary health care, health services accessibility

## Abstract

**Background**: Headache disorders, particularly migraine, are a leading cause of disability among active-duty military personnel, significantly affecting operational readiness and fitness for duty. Despite their high prevalence, limited data exist on how headache disorders are managed within military primary care systems. This study aimed to evaluate diagnostic confidence, treatment strategies, and structural challenges in the management of headache disorders from the perspective of military primary care physicians. **Methods**: A prospective, nationwide cross-sectional survey was conducted between May and July 2023 among all active-duty military physicians in primary care roles. An anonymous 15-item questionnaire assessed diagnostic practices, therapeutic approaches, referral pathways, perceived knowledge gaps, and suggestions for system improvements. The survey was distributed across military medical centers and outpatient clinics in Germany. **Results**: Ninety military physicians participated. Migraine and tension-type headache were commonly encountered, with 70% having treated at least one headache patient in the week prior to the survey. Diagnostic confidence was high for migraine (83.4%) and tension-type headache (77.8%) but lower for medication-overuse headache (65.5%) and cluster headache (47.8%). Acute treatment was widely implemented, but only 27.8% of respondents regularly initiated preventive therapies. Awareness of clinical guidelines was limited: only 23.3% were familiar with the ICHD-3, and just 58.9% with national headache treatment guidelines. Respondents expressed strong demand for targeted education, practical diagnostic tools, and improved interdisciplinary coordination. **Conclusions**: Headache disorders are a prevalent and clinically significant issue in military primary care. While military physicians show high engagement, important gaps exist in preventive treatment, guideline familiarity, and access to specialist care. Structured training, standardized treatment protocols, and system-level improvements are essential to optimize headache care and maintain operational readiness.

## 1. Introduction

Headache disorders—particularly migraine—represent an increasing challenge for military healthcare systems. Over the past two decades, numerous international studies have shown that migraine is not only one of the most frequently diagnosed neurological conditions in active-duty military personnel but also one of the most consequential in terms of service fitness, operational readiness, and long-term health outcomes [1,2,3,4,5,6,7,8,9,10,11,12,13,14,15,16,17,18,19,20,21,22,23,24].

Migraine and other headache disorders predominantly affect young adults, with the highest prevalence observed in the fourth decade of life [25,26,27,28,29,30]. Globally, migraine ranks second only to stroke among the leading neurological causes of years lived with disability (YLD) [21,29,30]. Across all age groups and sexes, it is the second most common cause of disability, and it is the leading cause among young women [24,31].

Within the military environment, migraine and other primary headache disorders significantly impair operational capability. Both retrospective and prospective studies have demonstrated that migraine contributes to a substantial proportion of medical disqualifications among aircrew and ground forces alike, surpassed only by mental health conditions such as depression and post-traumatic stress disorder [10,12,14]. In military medicine, it is not the formal diagnosis per se but rather factors such as persistent pain, chronic headache progression, and psychological comorbidities that are most strongly associated with increased duty limitations and medical disqualification [3,9,18].

Moreover, migraine occurs significantly more often in deployment contexts—particularly in combat settings—than in peacetime conditions. Combat exposure, sleep deprivation, hormonal fluctuations, stress, and traumatic brain injury act as potent triggers and pathophysiological cofactors contributing to the onset and chronification of the disorder [1,4,11]. Female service members are particularly affected, showing both higher prevalence rates and greater healthcare utilization than their male counterparts [1,2].

Given the high prevalence and operational impact of headache disorders among active-duty personnel, current treatment gaps pose not only a clinical challenge but a strategic threat to force readiness. Underdiagnosis, delayed interventions, and limited adherence to evidence-based guidelines undermine both individual health and long-term deployability [6,19,23]. This is particularly true for mission-relevant impairments such as high-frequency migraine attacks, chronic tension-type headaches, and post-traumatic headache syndromes. At the same time, data from military healthcare settings clearly demonstrate that early diagnosis and multidimensional treatment approaches—especially when addressing psychological comorbidities—can play a key role in preventing chronification and preserving service fitness [1,3,4,5,6,8,13,14,18,19,20,22,23,32,33,34,35]. Against this background, there is an urgent need to systematically improve headache care for service members. This includes not only clinical diagnostic and therapeutic processes, but also the organizational and structural frameworks of military healthcare systems.

The Bundeswehr constitutes the unified armed forces of Germany and currently comprises approximately 183,000 active-duty service members [36]. Medical care is provided through an extensive network of around 1000 military and civilian healthcare facilities, including five Bundeswehr hospitals. Primary care is predominantly delivered by military general practitioners, who serve as the first point of contact within the framework of free military healthcare services. Given the often non-specific symptom presentations, functional impairments, and frequent psychological comorbidities associated with headache disorders, military general practitioners play a key role in the early recognition, longitudinal monitoring, and coordinated management of migraine and other headache conditions in the armed forces.

The aim of this study is to systematically examine the key requirements, operational challenges, and structural gaps in the management of headache disorders among service members, from the perspective of frontline military healthcare providers. By conducting a nationwide cross-sectional survey, the study seeks to identify actionable fields for mission-oriented, resource-efficient, and guideline-based diagnostic, preventive, and therapeutic strategies within the military medical framework. The findings are intended to inform targeted improvements in clinical practice and healthcare planning, ultimately strengthening force health protection and long-term deployability.

## 2. Methods

### 2.1. Study Design and Population

This study was designed as a prospective cross-sectional cohort study using an anonymous online questionnaire. It formed part of a broader research initiative investigating the prevalence, diagnostic awareness, treatment strategies, and operational impact of headache disorders among active-duty personnel in the German Armed Forces (Bundeswehr). The survey was conducted between 15 May and 31 July 2023. The present analysis focuses specifically on the perspective of military primary care physicians (“Truppenärzte”) in order to assess the key requirements, operational challenges, and structural gaps in the management of headache disorders in the armed forces. The target population included all active-duty primary care physicians currently practicing within the Bundeswehr medical system.

### 2.2. Recruitment Strategy

Participant recruitment was initiated via an official information letter distributed to regional military medical units. This letter contained a study description, a data protection notice, and a direct link and QR code to facilitate low-threshold voluntary participation. Flyers were disseminated to seven regional medical support centers, 120 military outpatient clinics, and ten satellite medical facilities across Germany. Reminder emails were sent at weeks 7 and 9 following the initial invitation to increase participation rates.

### 2.3. Ethics Approval and Data Protection

The study was reviewed and approved as a designated research project by the Medical Service Command of the German Armed Forces (Project ID: 48K3-S-33 2323). Ethical approval was granted by the Ethics Committee of Kiel University (Reference: D 453/23). The study was conducted in accordance with the Declaration of Helsinki and applicable national data protection regulations. All participants were informed about the purpose, anonymity, and voluntary nature of the study prior to participation. Prior to participation, all respondents received detailed information about the aim, scope, voluntary nature, and data protection measures of the study. Informed consent was obtained electronically: participants were required to confirm their consent by actively ticking a checkbox before they could proceed to the questionnaire. No personal identifiers were collected, and data were analyzed in an anonymized form. Withdrawal after survey submission was not possible due to anonymization.

### 2.4. Instrument Development

The questionnaire consisted of 15 items covering personal demographics (e.g., age, specialty, years of clinical experience), recent headache case frequency, diagnostic confidence, therapeutic practices, and awareness of clinical guidelines. It also addressed non-pharmacological interventions, referral behavior, and preferences for future education and structural improvements. The survey included an optional free-text field for individual suggestions. To ensure content validity, the questionnaire was reviewed by a multidisciplinary panel of clinical experts in neurology, headache medicine, and military healthcare. Feedback was incorporated to enhance clarity, clinical relevance, and applicability to the military context. Psychometric evaluation (e.g., internal consistency, construct validity, test–retest reliability) was not part of the present study but is planned for future research. The use of an anonymous online questionnaire was chosen to maximize accessibility, ensure participant anonymity, and increase response rates across geographically dispersed units of the Bundeswehr. Given the sensitive nature of health-related disclosures—particularly in a military setting where concerns about stigma or career impact may inhibit reporting—an online, self-administered format likely encouraged more honest and complete responses compared to in-person interviews. Additionally, this method allowed for efficient large-scale data collection within a limited time frame and resource constraints.

### 2.5. Statistical Analysis

All survey data were collected and exported from the online survey platform Questionstar (https://www.questionstar.de, accessed on 5 March 2025) in Microsoft Excel format. Descriptive statistical analyses were performed using GraphPad Prism (Version 9.5.1) and RStudio (Posit PBC, Boston, MA, USA, Version 2024.09). Continuous variables are reported as means and standard deviations, while categorical variables are presented as absolute frequencies and percentages. Analyses were conducted based on complete case data; no imputation was performed for missing values.

## 3. Results

### 3.1. Demographic Data

A total of 90 military primary care physicians returned complete surveys. Table 1 presents their sociodemographic characteristics. More than half of the respondents (N = 49; 54.4%) were between 25 and 35 years old; over 90% were under the age of 45. Approximately one-third had less than five years of professional experience, and another third had five to ten years. Around two-thirds of the participants were either board-certified in general medicine or in advanced training to become general practitioners. The remaining third included physicians in training from other specialties such as internal medicine, anesthesiology, psychiatry, surgery, urology, dermatology, otolaryngology, and neurosurgery. No participants were in training to become neurologists.

### 3.2. Headache in Military Primary Care Settings

Seventy percent of respondents reported having seen 1–5 patients with a headache in the week prior to the survey; approximately 13% reported 6–10 headache patients, around 3% reported 11–20, and 13% had not seen any headache patients that week. Regarding the estimated proportion of headache cases in their daily practice, 34.4% of primary care physicians estimated the share to be less than 5%, 38.9% around 5%, 15.6% around 10%, 7.8% around 15%, and 3.3% around 20% (Table 1).

Diagnostic confidence was high for migraine (83.4%) and tension-type headache (77.8%). Confidence was lower for medication-overuse headache (65.5%), and fewer than half of respondents felt confident diagnosing cluster headache (47.8%) (Table 2).

### 3.3. Treatment Approaches

Table 3 displays the findings on the treatment practices. Two-thirds (66.7%) of respondents reported that they always or often advised patients to keep a headache diary. Neuroimaging or referrals to neurologists or headache specialists were handled variably. Psychiatric comorbidities were routinely assessed by 70% of physicians, and sleep quality by 67.8%.

**Figure 1:**
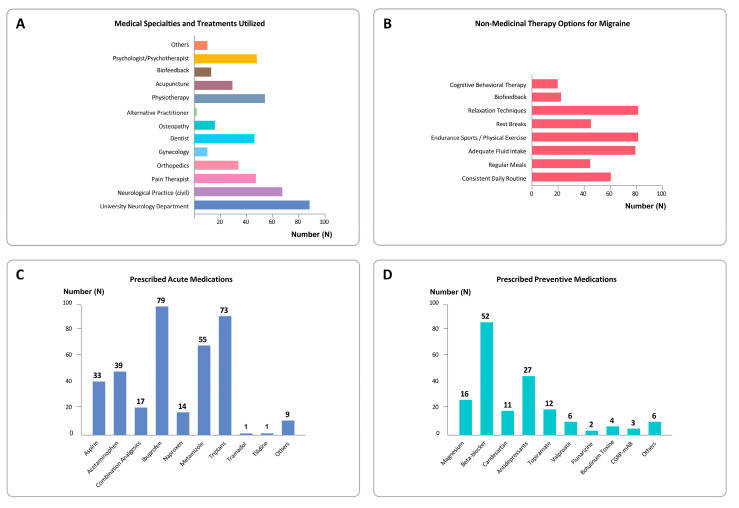
Treatment of headache patients in military primary care. (**A**) shows the medical specialties and therapeutic options considered by military physicians for the treatment of migraine. (**B**) illustrates which non-pharmacological recommendations are routinely discussed with migraine patients. (**C**) provides an overview of the medications prescribed for acute migraine attacks. (**D**) displays the pharmacological agents prescribed for migraine prophylaxis.

Regarding referrals and adjunct treatments for patients with migraine, the most frequently named options were military neurological evaluation units and civilian neurologists, followed by physiotherapy, psychotherapy, pain specialists, dentists, and orthopedics. Acupuncture and osteopathy were mentioned more often than biofeedback (Figure 1A).

Over three-quarters (76.6%) of respondents stated that they always (42.2%) or often (34.4%) informed patients about the risk of medication-overuse headache. Non-pharmacological treatment options were always or often discussed by 81.1% of primary care physicians. For migraine, these included hydration, aerobic exercise, and relaxation techniques (Figure 1B).

Ibuprofen was the most commonly reported acute medication used for migraine management, followed by triptans and metamizole. Other medications included acetylsalicylic acid, paracetamol, combination analgesics, and naproxen. Opioids such as tilidine and tramadol were also reported. Antiemetics such as metoclopramide were used as adjuncts (Figure 1C).

In contrast, only 27.8% of respondents reported that they always or often initiated prophylactic treatment in patients with frequent or disabling headaches or migraine. The most commonly used preventive medications were beta-blockers, followed by antidepressants and magnesium (Figure 1D).

### 3.4. Guidelines and Continuing Education

As shown in Table 4, the most frequently used sources of information on headache and migraine were peer discussions and medical online platforms. A large majority (92.2%) of respondents expressed a desire for more continuing education on headache and migraine (Table 4). Only 21 respondents (23.3%) reported being familiar with the International Classification of Headache Disorders, 3rd edition (ICHD-3). Fifty-three primary care physicians (58%) were aware of the national guidelines issued by the German Society of Neurology (DGN) for migraine, tension-type headache, cluster headache, and medication-overuse headache (Table 4).

### 3.5. Suggestions for Improvement

The final open-ended question regarding suggestions for improving the care of headache patients in military practice was answered by 43 respondents (47.8%). A total of 58 distinct statements were extracted and categorized (Table 5). More than a quarter (27.9%) called for concrete clinical guidance for military primary care physicians, in the form of concise standard operating procedures (SOPs). The respondents stated that these SOPs should include diagnostic criteria for various headache types, stepwise pharmacologic treatment algorithms, and clear indications for imaging or referral to specialists. Additional suggestions included providing headache diaries and patient information materials for soldiers, enhancing training opportunities for Truppenärzte, and improving coordination with military neurology units—particularly regarding appointment availability, accessibility, and the designation of specific contact persons. Furthermore, 9.3% of respondents stated that they would welcome more time for the management of headache patients. Approximately 7% supported the establishment of specialized outpatient clinics for headache, the use of standardized questionnaires for history-taking, and the expansion of non-pharmacological treatment options at Bundeswehr medical facilities.

## 4. Discussion

This study provides the first systematic insight into headache care within the primary medical service of the German Armed Forces. Our findings confirm that military primary care physicians regard headache disorders—particularly migraine and tension-type headache—as clinically relevant and frequently encountered in practice. This is consistent with international literature reporting high prevalence rates of headache disorders and their considerable impact on operational capacity and healthcare utilization in military populations [10,19,23,37].

Despite high diagnostic confidence for common primary headache types, substantial uncertainty remains regarding less frequent and more complex conditions such as cluster headache and medication-overuse headache. Similar diagnostic challenges have been noted in studies of post-traumatic headache (PTH), where poor phenotypic differentiation often hampers treatment planning [9,13]. Finkel et al. [9], for example, found that continuous pain patterns—rather than diagnostic labels—were more predictive of occupational impairment in service members with mild traumatic brain injury (mTBI)-related headache.

The limited use of preventive treatment options observed in our cohort (only 27.8% of physicians initiate prophylaxis regularly) echoes findings from U.S. and NATO-aligned forces, where migraine and other primary headaches remain frequently undertreated [12,18]. Reasons may include diagnostic hesitation, lack of familiarity with current guidelines, and logistical constraints such as limited time, staffing shortages, and poor continuity of care between primary and specialist services.

At the same time, the routine use of headache diaries and screening for psychiatric comorbidities and sleep disturbances by a majority of respondents reflects a commendable awareness of the biopsychosocial dimensions of chronic headache. This aligns with findings from Baker et al. [3], who demonstrated that comorbid depression was a stronger predictor of duty limitations than headache severity itself, yet often remained underdiagnosed and undertreated in military contexts.

The strong demand for continuing education (92.2% of respondents) underscores both the relevance of the topic and the readiness among military physicians to improve headache care. Respondents expressed a clear preference for practice-oriented formats such as SOPs, treatment algorithms, and interdisciplinary case discussions. This supports calls from international societies for the implementation of scalable, evidence-based training structures that address headache care, in general, in medical settings [38,39].

Furthermore, several respondents reported insufficient access to specialist neurology services and weak coordination mechanisms, particularly in deployment contexts. These structural deficits have been described previously and highlight the need for telemedicine expansion, designated neurology liaison officers, and improved referral pathways [5,6,7,38,39].

Taken together, our findings demonstrate that although basic awareness and interest in headache care are present among Bundeswehr primary care providers, key deficits persist in diagnostic precision, preventive treatment, access to guidelines, and interdisciplinary coordination. These deficits mirror international experience but require tailored solutions that account for the specific organizational and operational framework of the German Armed Forces.

We recommend that headache care be formally recognized as a core competency in military medicine, with structured training and standardized tools embedded in both pre- and postgraduate medical education. Given the high burden and operational relevance of headache disorders, military healthcare planning should include provisions for clinical upskilling, diagnostic standardization, and access to interdisciplinary care.

### 4.1. Limitations

This study has several limitations. The voluntary nature of participation may have introduced selection bias, favoring more engaged or experienced respondents. In addition, the results are based on self-reported data and, therefore, subject to potential recall and reporting bias. Moreover, clinical outcome measures, real-world care pathways, and longitudinal follow-up data were not assessed. Finally, while content validation of the questionnaire was performed, psychometric validation remains pending and should be addressed in future studies.

### 4.2. Implications

The results highlight multiple leverage points for improving the diagnosis and treatment of headache disorders within the Bundeswehr medical service (Table 6). First, given the substantial prevalence and impact on fitness for duty, headache care should be formally recognized as a core competency in military medicine. This includes integrating headache management into medical curricula and ongoing training structures.

Second, while diagnostic confidence is high for migraine and tension-type headache, the marked uncertainty in diagnosing cluster headache and medication-overuse headache underscores the need for targeted CME modules covering rare and secondary headache forms.

Third, the fact that only 27.8% of physicians initiate preventive treatment regularly points to a therapeutic gap. To address this, preventive strategies should be promoted through clearly defined standard operating procedures (SOPs) and algorithm-based treatment pathways, ideally supported by digital platforms.

Fourth, the limited familiarity with key classification systems indicates that guideline-based training must be implemented more systematically and at scale. Ensuring practical applicability in routine care should be a priority.

Fifth, the pronounced interest in further education (92.2%) reflects a strong willingness among military primary care physicians to engage in quality improvement. This should be leveraged through continuous, accessible, and practice-oriented training formats.

Sixth, structural and organizational barriers—particularly the lack of access to neurology units and weak coordination mechanisms—should be addressed by improving referral pathways, designating neurology liaison contacts, and expanding telemedicine capabilities, both in garrison and in deployment contexts.

Seventh, multiple calls for operational tools such as SOPs, headache diaries, and patient education materials demonstrate the necessity of developing standardized resources to support frontline headache care.

Eighth and finally, broader systemic constraints—including limited time, personnel shortages, and lack of access to non-pharmacological therapies—must be acknowledged in health policy decisions. Headache care should be strategically integrated into military health planning, resource allocation, and force health protection frameworks.

## 5. Conclusions

Headache disorders, particularly migraine, constitute a clinically relevant and operationally impactful condition among service members of the German Armed Forces. This study underscores the pivotal role of military general practitioners in early recognition, frontline management, and coordinated referral. To safeguard mission capability and long-term health, the integration of headache medicine into military medical training, clinical SOPs, and health system structures is essential. Future research should focus on longitudinal outcomes, quality-of-care indicators, and digital support systems to strengthen continuity of care and improve functional outcomes for soldiers with headache disorders.

## Figures and Tables

**Table 1:** Overview of the characteristics of the study population.

		N	%
Age	25–35 years	49	54.4
	36–45 years	34	37.8
	46–55 years	5	5.6
	56–65 years	2	2.2
Specialization	General medicine	60	66.7
	Internal medicine	12	13.3
	Anesthesia	4	4.4
	Psychiatry	3	3.3
	General surgery	2	2.2
	Urology	2	2.2
	Dermatology	2	2.2
	Orthopedics/trauma surgery	1	1.1
	ENT	1	1.1
	Neurosurgery	1	1.1
	Other	2	2.2
Professional experience	<5 years	32	35.5
	5–10 years	31	34.4
	11–15 years	13	14.4
	16–20 years	7	7.8
	21–25 years	4	4.4
	>25 years	3	3.3
Number of headache patients last week	0	12	13.3
	1–5	63	70.0
	6–10	12	13.3
	11–15	1	1.1
	16–20	2	2.2
	>20	0	0
Estimated proportion of headache patients in military medical consultations	<5%	31	34.4
	About 5%	35	38.9
	About 10%	14	15.6
	About 15%	7	7.8
	About 20%	3	3.3
	>20%	0	0

**Table 2:** Certainty of diagnosis.

	Migraine		Tension-Type Headache		Cluster Headache		Medication-Overuse Headache	
	N	%	N	%	N	%	N	%
Very safe	7	7.8	17	18.9	10	11.1	10	11.1
Rather safe	68	75.6	53	58.9	33	36.7	49	54.4
Rather unsafe	14	15.6	20	22.2	42	46.7	27	30.0
Very unsafe	1	1.1	0	0	5	5.6	4	4.4

**Table 3:** Treatment practice for headache patients.

	Always		Often		Sometimes		Rarely		Never	
	N	%	N	%	N	%	N	%	N	%
Do you order imaging studies for your headache patients?	7	7.8	21	23.3	42	46.7	19	21.1	1	1.1
Do you assess for psychological comorbidities such as depression or anxiety disorders?	36	40.0	27	30.0	13	14.4	12	13.3	2	2.2
Do you inquire about sleep quality in your headache patients?	33	36.7	28	31.1	18	20.0	11	12.2	0	0
Do you ask your headache patients to keep a headache diary?	34	37.8	26	28.9	17	18.9	9	10.0	4	4.4
Do you refer your headache patients to a neurologist, pain specialist, or headache expert?	3	3.3	35	38.9	46	51.1	5	5.6	1	1.1
Do you educate patients with migraine or tension-type headache about non-pharmacological treatment options?	45	50.0	28	31.1	12	13.3	4	4.4	1	1.1
Do you offer preventive pharmacological treatment to patients with frequent or disabling headaches or migraine?	6	6.7	19	21.1	25	27.8	24	26.7	16	17.8
Do you inform patients about the risk of medication overuse or medication-overuse headache?	38	42.2	31	34.4	10	11.1	9	10.0	2	2.2

**Table 4:** Sources of information on headaches and migraines.

Where Do You Get Your Information on Headaches and Migraines?	N	%
Textbooks	39	43.3
Medical journals	30	33.3
International Classification of Headache Disorders (ICHD-3)	10	11.1
Guidelines of the neurological society DGN	33	36.7
Homepage of the Migraine and Headache Society DMKG	5	5.6
Medical online platforms	59	65.6
Exchange with colleagues	67	74.4
Other	8	8.9
Guidelines and continuing education		
I am familiar with the International Classification of Headache Disorders (ICHD-3).	21	23.3
I am familiar with the guidelines of the German Society of Neurology on migraine, tension-type headache, cluster headache, and medication-overuse headache.	53	58.9
I would like to receive more training and continuing education on migraine and headache disorders for my work as a military physician.	83	92.2

**Table 5:** Open-ended question on suggestions for improving the treatment of headache patients in military primary care consultations.

Statements (N = 58)	N	%
Concrete clinical recommendations for military physicians/SOPs	12	20.7
Headache diary for service members	6	10.3
More training and continuing education for military physicians	5	8.6
Improved collaboration with neurological evaluation units (accessibility, appointment scheduling, designated contact persons)	5	8.6
Flyers/informational materials for headache patients	5	8.6
More time allocated for headache consultations	4	6.9
Specialized outpatient clinic/dedicated headache consultation	3	5.2
Standardized questionnaires to facilitate medical history taking	3	5.2
Improved access to non-pharmacological treatment options at military sites	3	5.2
Interdisciplinary collaboration, e.g., with dental services	2	3.4
Reduced physician turnover, increased staffing	2	3.4
App-based digital health application for headache patients	2	3.4
Reduction in administrative barriers in medication procurement	2	3.4
Communication tips for consultations with headache patients	1	1.7
Online informational events for headache patients	1	1.7
Structured treatment programs specifically for headache patients	1	1.7
Free access to alternative therapies such as osteopathy or acupuncture	1	1.7

**Table 6:** Implications for military primary headache care.

Key Findings	Implications for Military Health Policy
High prevalence of headache disorders in military primary care	Headache care should be recognized as a core competency in military medicine
High diagnostic confidence for migraine and tension-type headache	Training programs are effective, but need expansion to cover complex headache types
Low confidence in diagnosing cluster headache and medication-overuse headache	Include focused modules on rare/secondary headaches in military CME programs
Only 27.8% of physicians initiate preventive treatment regularly	Promote preventive strategies through SOPs and treatment algorithms
Only 23.3% are familiar with ICHD-3, 58% with national guidelines	Systematic implementation of guideline-based training is needed
Strong interest (92.2%) in further education on headache care	Establish continuous, accessible, headache-focused training formats
Lack of access to neurology units and insufficient coordination reported	Improve referral pathways, designate neurology liaisons, and expand telemedicine capabilities
Multiple calls for SOPs, headache diaries, and patient education materials	Develop standardized tools and resources for frontline care
Structural barriers: limited time, staffing, and availability of non-pharma care	Integrate headache care into military health planning and resource allocation

## Data Availability

The original data presented in the study are openly available in Zenodo https://doi.org/10.5281/zenodo.15501639.
